# Investigating the therapeutic profile of velaglucerase alfa in paediatric patients with Gaucher disease: a systematic review across all paediatric age groups

**DOI:** 10.1186/s13023-026-04221-9

**Published:** 2026-02-05

**Authors:** Javier de las Heras, Jorge J Cebolla, Sofía de Pedro, Manuel Gómez-Barrera, Isidro Vitoria

**Affiliations:** 1 Division of Paediatric Metabolism, Cruces University Hospital, CIBER-ER, Metab-ERN, University of the Basque Country (UPV/EHU),, Biobizkaia Health Research Institute, Bilbao, Spain; 2 Takeda Farmacéutica España S.A, Calle Albacete 5, 9th floor- Madrid, Madrid, Spain; 3 Pharmacoeconomics & Outcomes Research Iberia (PORIB), Madrid, Spain; 4 Unit of Metabolopathies and Nutrition, University Hospital, La Fe, Valencia, Spain

**Keywords:** Type 1 Gaucher disease, Type 3 Gaucher disease, Enzyme replacement therapy, Velaglucerase alfa, Paediatric patients, All ages, Home therapy, Quality of life

## Abstract

**Background:**

Gaucher disease (GD) is a rare autosomal recessive genetic disorder. The clinical manifestations can be adequately managed with enzyme replacement therapy (ERT). The aim of this systematic literature review was to explore the safety and efficacy or effectiveness (depending on the type of evidence) profile of velaglucerase alfa in the treatment of paediatric patients with type 1 (GD1) and type 3 (GD3) GD across all paediatric ages.

**Methods:**

A systematic review of the PubMed/Medline and Embase databases, along with communications from international conferences, was conducted. The inclusion criteria comprised clinical studies published in either English or Spanish that assessed the therapeutic profile of velaglucerase alfa in patients with GD1 (primarily) and GD3 (exploratorily) of all paediatric ages (0–18 years). For each of the selected publications, data regarding the safety and efficacy/effectiveness of this treatment were extracted.

**Results:**

A total of 539 publications were identified, of which 23 studies encompassing data from 159 paediatric patients were included. Nine studies (71 patients) provided information about the safety in paediatric patients with GD1, describing it as well tolerated. Regarding the efficacy/effectiveness, 14 articles (113 patients) reported relevant data for the same subpopulation. Overall, improvements in haematological, visceral, skeletal, biomarker and health-related quality-of-life outcomes have been described in treatment-naïve paediatric patients with GD1 who were initially treated with velaglucerase alfa, as well as maintained stability in patients previously treated with imiglucerase. Furthermore, it has been reported that the safety and efficacy/effectiveness profile administered as home therapy enhances the quality of life for both patients and caregivers. The use of velaglucerase alfa in paediatric patients with GD3 was described in 7 publications (26 patients), suggesting a favourable safety profile, whereas its efficacy/effectiveness was reported in 5 articles (16 patients). Improvements in the non-neurological manifestations of the disease were recorded in patients with GD3.

**Conclusion:**

This systematic review summarizes the limited evidence on velaglucerase alfa in paediatric patients with GD. Findings suggest that velaglucerase alfa may be a beneficial option for GD1 across all paediatric age groups (0–18 years). Additionally, it might be considered a therapeutic option for non-neurological GD3 symptoms, although evidence is scarce and exploratory, highlighting the need for further research in those patients.

**Supplementary Information:**

The online version contains supplementary material available at 10.1186/s13023-026-04221-9.

## Background

Gaucher disease (GD; ORPHA:355), recognized as one of the most common lysosomal diseases, is an infrequent autosomal recessive genetic disorder. The estimated prevalence of GD worldwide varies between 0.02 and 139.0 per 100,000 individuals [1], with an anticipated incidence rate of 1 per 40,000–60,000 individuals annually [2]. The disease originates from pathogenic mutations in the *GBA1* gene (MIM*606463), leading to a deficiency of the enzyme β-glucosidase (GCase; EC.3.2.1.45) [2]. Due to the diminished activity of the enzyme GCase [2], this disease leads to an accumulation of its substrate, glucosylceramide (GluCer), as well as its deacylated form, glucosylsphingosine (Lyso-Gb1), primarily within the lysosomes of the monocyte-macrophage lineage.

The disease is categorized into three primary phenotypes according to the presence of neurological involvement, the age of onset, and the rate of disease progression [4, 5]. Type 1 GD (GD1; MIM#230800; ORPHA:77259), also known as non-neurological GD, is the most prevalent type of GD in Western countries, accounting for more than 90% of cases [6]. Type 2 GD (GD2; MIM#230900; ORPHA:77260), known as acute neurological GD (nGD), has an early onset and a rapidly progressive course, often leading to death within the first few years of life [2, 3]. Type 3 GD (GD3; MIM#231000; ORPHA:77261), referred to as chronic nGD, typically manifests during childhood or adolescence and has a slower neurological progression, with some patients developing visceral and/or skeletal involvement [2, 3].

For the treatment of specific manifestations of the disease, two approaches are currently available: substrate reduction therapy (SRT) and enzyme replacement therapy (ERT) [7, 8]. ERT is currently the standard of care for paediatric patients with GD. It has the potential to alleviate or even eliminate the symptoms of the disease [5]. Additionally, it can prevent further complications and improve the quality of life (QoL) of patients [5]. However, it is important to note that neither of these treatments is effective in treating the neurological symptoms present in patients with nGD. This is because these treatments do not cross the blood-brain-barrier (BBB) or because their distribution within the brain is effectively null [5, 9].

Currently, in Europe, all SRT approaches for the treatment of paediatric patients with GD are not authorised for this population and those with authorisation carry label restrictions mainly related to age, weight, and pharmacogenomic factors [10–13], with ERT remaining the only fully labelled option indicated for this subpopulation. Two ERT molecules are available for the treatment of paediatric patients with GD: (1) Imiglucerase, which is produced by recombinant DNA technology using a mammalian chinese hamster ovary cell culture, is indicated as a long-term therapy for all patients with GD1 and for patients with GD3 with clinically relevant, non-neurological manifestations of the disease [14]. (2) Velaglucerase alfa, a gene-activated molecule produced in a human cell line with the same amino acid sequence as the naturally occurring human GCase, is indicated as a long-term therapy for patients with GD1 [15]. Velaglucerase alfa is also sometimes used to treat the non-neurological manifestations of GD in patients with GD3 [17].

For patients with GD, the initiation of specific treatment early in the disease course has been associated with better long-term clinical outcomes [5, 16, 18, 19]. This recommendation is particularly relevant in childhood, as the disease, if left untreated for prolonged periods of time, progresses with increasing rapidity and severity compared with that in adulthood. Hence, starting treatment as soon as possible in all children and teenagers with GD is recommended [18, 20]. Several studies focusing on the treatment of paediatric patients with GD1 with velaglucerase alfa have reported satisfactory outcomes [5]. However, the safety and efficacy profile of this treatment in patients with GD1 younger than 4 y have not yet been established within the framework of clinical trials. Furthermore, there is a lack of synthesized information on the administration of velaglucerase alfa in patients with GD1 across all paediatric age groups (0–18 y), as well as in patients with GD3, based on “real-world experience”. The systematic literature review methodology may help address certain gaps related to the treatment of paediatric patients with GD1 across all paediatric ages (0–18 y) and explore those related to patients with GD3 using velaglucerase alfa.

The primary aim of the current investigation was to conduct a systematic review of the available literature to collate and summarize the findings pertaining to the safety and efficacy or effectiveness (depending on the type of evidence) profile of velaglucerase alfa in the therapeutic intervention of paediatric patients diagnosed with GD1, irrespective of their age. Concurrently, as a exploratory aim, the study also sought to examine the safety and efficacy or effectiveness profile of velaglucerase alfa in the therapeutic intervention of non-neurological manifestations in paediatric patients with GD3.

## Methods

### Design of the review

This systematic review was conducted by a multidisciplinary team composed of paediatricians with comprehensive expertise and experience in the management of GD, as well as specialists in the development of literature reviews and health data analysis.

The review protocol was formulated in compliance with the Preferred Reporting Items for Systematic Reviews and Meta-Analyses (PRISMA) guidelines [21]. The search strategy was designed following the PICO (population, interventions, comparisons, outcomes) framework: population of interest (patients with GD across all paediatric ages, 0–18 y), intervention(s) (velaglucerase alfa), comparison(s) (unrestricted), and outcomes (safety and efficacy/effectiveness profiles). This protocol underwent a thorough review and received approval from the entire team.

### Data sources and search strategy

A literature search strategy employing various descriptors related to the treatment of GD (“Gaucher”, “Disease”, “Velaglucerase”, and “VPRIV”) was devised (October 19, 2023) to identify relevant studies (Table 1).

**Table 1:** PubMed/Medline and embase search strategy

Terms		Search strategy Search date: October 19, 2023
**POPULATION**		
#1	Gaucher	“Gaucher“[Title/Abstract]
#2	Disease	“disease“[Title/Abstract]
#3	#1 AND #2	“Gaucher“[Title/Abstract] AND “disease“[Title/Abstract]
**INTERVENTION**		
#4	Velaglucerase	“velaglucerase“[All Fields]
#5	VPRIV	“velaglucerase alfa“[All Fields] OR “vpriv“[All Fields]
#6	#4 OR #5	“velaglucerase“[All Fields] OR “velaglucerase alfa“[All Fields] OR “vpriv“[All Fields]
**POPULATION + INTERVENTION**		
#7	#3 AND #6	“Gaucher“[Title/Abstract] AND “disease“[Title/Abstract] AND (“velaglucerase“[All Fields] OR (“velaglucerase alfa“[All Fields] OR “vpriv“[All Fields]))

The search encompassed the PubMed/Medline and Embase databases. In addition, a manual search was undertaken to identify studies presented at a variety of well-recognized international conferences on inborn error of metabolism disorders. These included the Society for the Study of Inborn Errors of Metabolism (SSIEM), the International Congress of Inborn Errors of Metabolism (ICIEM), the WORLD*Symposium*, and the Society for Inherited Metabolic Disorders (SIMD) [22–24]. The search strategy for PubMed/Medline was developed, agreed upon, and subsequently adapted for Embase and the manual search. There was no restriction by year of publication, except for communications presented at conferences. For the latter, the search was limited to the last 10 y (2013–2023).

### Study selection and data extraction

All studies involving patients with GD that incorporated velaglucerase alfa as an intervention and were conducted in paediatric patients (0–18 y) were included in the analysis. Furthermore, eligible records needed to be grounded in clinical studies such as clinical trials, case series, cohort studies, or meta-analyses and had to be published in either English or Spanish. Conversely, studies were excluded if they described pathologies other than GD, if they did not include velaglucerase alfa as an intervention, or if they were conducted in adult patients (≥ 18 y). Moreover, publications that were not based on clinical studies such as reviews, editorials or preclinical studies and articles published in any language other than English or Spanish were also excluded.

Each citation obtained was independently reviewed by two of the authors (MGB and SPM) against the eligibility criteria in a two-stage process: (1) title and abstract and (2) full text. During the study selection and data extraction stages, any disagreements were resolved through discussion or with a decision made by a third researcher.

For the extraction of information from all the selected publications, a data matrix was constructed in Microsoft Excel^®^ in accordance with the PRISMA 2020 guidelines [21]. From each article, data pertaining to the following study characteristics were extracted: type of publication, country where the study was conducted, type and duration of the study, number and age of patients, type of GD, and treatment status (treatment-naïve vs. previously treated patients). In addition, data on safety and efficacy/effectiveness were also collected. The safety endpoints extracted included treatment emergent adverse events (TEAEs) and immunogenicity data (anti-velaglucerase alfa antibodies (ADAs)). The efficacy/effectiveness endpoints evaluated included haematological parameters (concentration of haemoglobin (Hb) and platelet counts), visceral parameters (volumes of liver and/or spleen), and skeletal parameters (bone marrow infiltration, bone mineral density (BMD), skeletal events, and growth in weight and height). The plasma biomarkers assessed included chitotriosidase (ChT), chemokine (C–C motif) ligand 18 (CCL18)/pulmonary and activation-regulated chemokine (PARC) (CCL18/PARC), and Lyso-Gb1. Developmental milestones, health-related quality of life (HRQoL) parameters (the 36-Item Short Form Health Survey (SF-36) and the Pediatric Quality of Life Inventory (PedsQL)), and the administration of velaglucerase alfa at home were also evaluated.

## Results

**Fig. 1:**
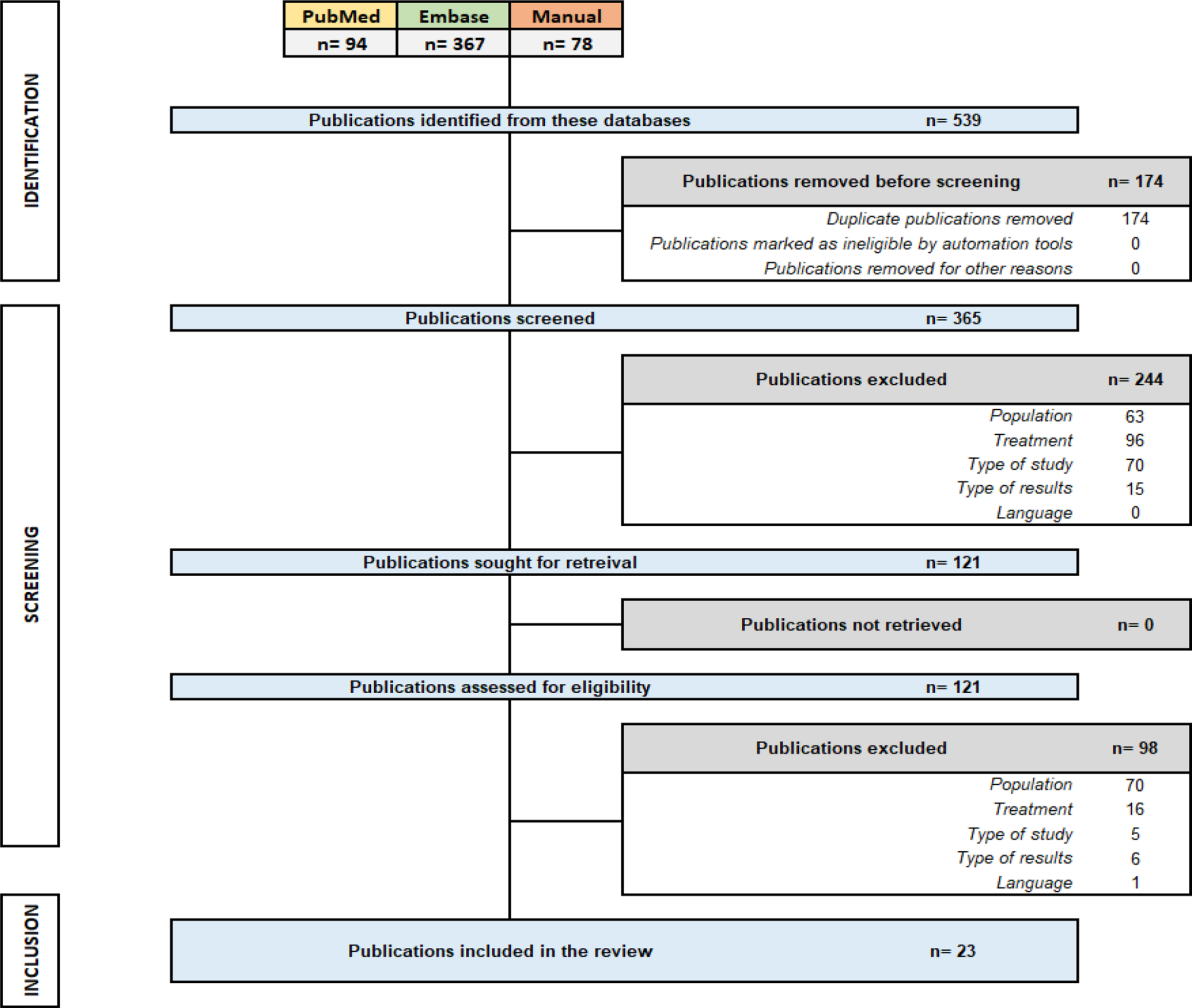
PRISMA flow chart of the publication selection process

A comprehensive search yielded a total of 539 publications: 94 were identified in PubMed/Medline, 367 were found in Embase, and an additional 78 were manually retrieved from international conference proceedings. After duplicates were removed, 365 articles remained for evaluation. An initial review of the titles and abstracts led to the exclusion of 244 publications. The subsequent full-text examination of the remaining 121 articles resulted in the exclusion of an additional 98 articles. Consequently, data were extracted from a total of 23 publications (Fig. 1).

### Characteristics of the studies and patients

Among the 23 publications, 13 (56.52%) were full-text articles [25–37], whereas the remaining 10 (43.48%) were communications presented at various conferences [38–47]. With respect to the study design, 13 publications (56.52%) were categorized as clinical trials [26, 30–32, 34, 35, 37, 40, 41, 44–47], 5 (21.74%) as observational studies [25, 29, 33, 38, 43], and the remaining 5 (21.74%) as case studies [27, 28, 36, 39, 42]. Patients with GD1 were described in 15 publications (65.22%) [26, 27, 31–35, 37, 40, 42–47], whereas those with GD3 were described in 7 publications (30.43%) [29, 30, 32, 33, 36, 41, 43]. Four studies (17.39%) did not specify the type of GD [25, 28, 38, 39]. Details about the studies that did not specify the type of GD are shown in the supplementary material (Table S1). There was considerable heterogeneity in the countries in which the different studies were conducted (*n* = 17 countries).

In total, the studies assessed included 156 patients ranging in age from 6 weeks to 17 y. Among these patients, 114 (73.08%) were diagnosed with GD1, and 26 (16.67%) were diagnosed with GD3. The type of GD was not specified for 16 patients (10.26%). With respect to treatment status, 80 patients (51.28%) were naïve, whereas 57 (36.54%) were patients previously treated with imiglucerase. The treatment status was not described for 19 patients (12.18%).

The main characteristics of the studies and patients included in this literature review are summarized in Table 2.

**Table 2:** Main characteristics of the studies in paediatric patients with GD included in the systematic review

Author, year [reference]	Type of publication	Country	Type of study^1^	Duration of the study	Number of patients (age)	Type of GD	Treatment status (number of patients)
Basiri, 2023 [25]	Article	USA	Longitudinal retrospective	20 y	2 (ND)	ND	N (2)
Becker-Cohen, 2023 [26]	Article	Israel	Prospective clinical trial (NCT03702361)	24 months	1 (10 y)	1	N (1)
Goker-Alpan, 2023 [38]	Abstract	USA	Ambispective phase 4 observational (NCT04721366)	ND	12 (3– to 71 months)	ND	ND
Stiles, 2021 [27]	Article	USA	Case	3 months	1 (7 y)	1	N (1)
Soudek, 2020 [28]	Article	Canada	Case	8.5 months	1 (6 weeks)	ND	N (1)
Soudek, 2019 [39]	Abstract	Canada	Case	2 months	1 (6 weeks)	ND	N (1)
Schwartz, 2018 [29]	Article	Multiple	*Gaucher* O*utcome Survey* (NCT03291223)^2^	5 y	9 (ND)	3	N (5) and PTP (4)
Tantawy, 2018 [30]	Article	Multiple	Phase 1/2 clinical trial (NCT01685216)	12 months	6 (2– to 14 y)	3	N (6)
Zimran, 2018 [31]	Article	Multiple	Phase 3 clinical trials and extension (NCT00635427, NCT00430625, NCT00553631)	4 y	8 (6– to 16 y)	1	N (8)
Elstein, 2016 [40]	Abstract	Multiple	Post hoc study of the extension of the NCT00478647 and NCT00553631 clinical trials (NCT00635427)	4 y	15 (ND)	1	N (15)
Ida, 2016 [32]	Article	Japan	Extension of the NCT01614574 clinical trial (NCT01842841)	12 + 12 months^3^	3 (11 to − 15 y)	1 and 3	PTP (3)
Pastores, 2016 [33]	Article	ND	Retrospective observational (includes 10 clinical trials)	11 y	2 (3 to 12 y)	1 and 3	PTP GD1 (1) and N GD3 (1)
Smith, 2016 [34]	Article	Multiple	Extension of the NCT00430625, NCT00478647 and NCT00553631 clinical trials (NCT00635427)	4 y	24 (3– to 16 y)	1	N (10) and PTP (14)
Tantawy, 2016 [41]	Abstract	Multiple	Phase 1/2 clinical trial (NCT01685216)	12 months	6 (2– to 14 y)	3	N (6)
Bailey, 2015 [42]	Abstract	USA	Case	12 months	1 (15 y)	1	PTP (1)^4^
Pastores, 2015 [43]	Abstract	ND	Retrospective observational (includes 8 clinical trials)^5^	7 y	2 (3– to 13 y)	1 and 3	PTP GD1 (1) and N GD3 (1)
Pastores, 2014 [44]	Abstract	Multiple	Extension of the NCT00430625, NCT00478647 and NCT00553631 clinical trials (NCT00635427)	4 y	24 (4– to 17 y)	1	N (10) and PTP (14)
Giraldo, 2013 [45]	Abstract	Multiple	Subgroup analysis of a phase 2/3 clinical trial and its extension (NCT00478647 and NCT00635427)	4 y (1 + 3)^6^	9 (9– to 16 y)	1	PTP (9)
Turkia, 2013 [35]	Article	Multiple	Phase 3 noninferiority, randomized, double-blind clinical trial (NCT00553631)	9 months	4 (7– to 14 y)	1	N (4)
Vairo, 2013 [36]	Article	Brazil	Case	12 y	1 (14 y)	3	PTP (1)
Zimran, 2013 [46]	Abstract	Multiple	Clinical trial and extension (NCT00553631 and NCT00635427)	5 y	8 (6– to 16 y)	1	N (8)
Zimran, 2013 [37]	Article	Multiple	Phase 2/3 clinical trial (NCT00478647)	2 y	9 (9– to 16 y)	1	PTP (9)
Zimran, 2010 [47]	Abstract	Multiple	Subgroup analysis of a phase 3 randomized, double-blind clinical trial^5^	ND	7 (ND)	1	ND

GD: Gaucher disease; N: Treatment-naïve; ND: No data; PTP: Patient previously treated with imiglucerase

^1^ All clinical trials and their extensions can be accessed on the ClinicalTrials.gov website: https://www.clinicaltrials.gov/

^2^*Gaucher Outcome Survey* (GOS) is an ongoing, international, observational, long-term disease-specific registry established in 2010 and sponsored by Shire a Takeda company (https://clinicaltrials.gov/ct2/show/NCT03291223) for patients with a confirmed Gaucher disease diagnosis, regardless of treatment status or type of treatment received, and who are not actively participating in a clinical trial for Gaucher disease

^3^ Clinical trial of 12 months, followed by an extension of another 12 months

^4^ Patient previously treated with imiglucerase and then treated with velaglucerase alfa alone and then in combination with eliglustat

^5^ The abstract does not specify the clinical trials that were considered in the analysis (i.e., no clinical trials’ identifiers were reported)

^6^ Clinical trial of 1 y, followed by an extension of 3 y

### Safety profile of velaglucerase Alfa in the treatment of paediatric patients with GD1

**Fig. 2:**
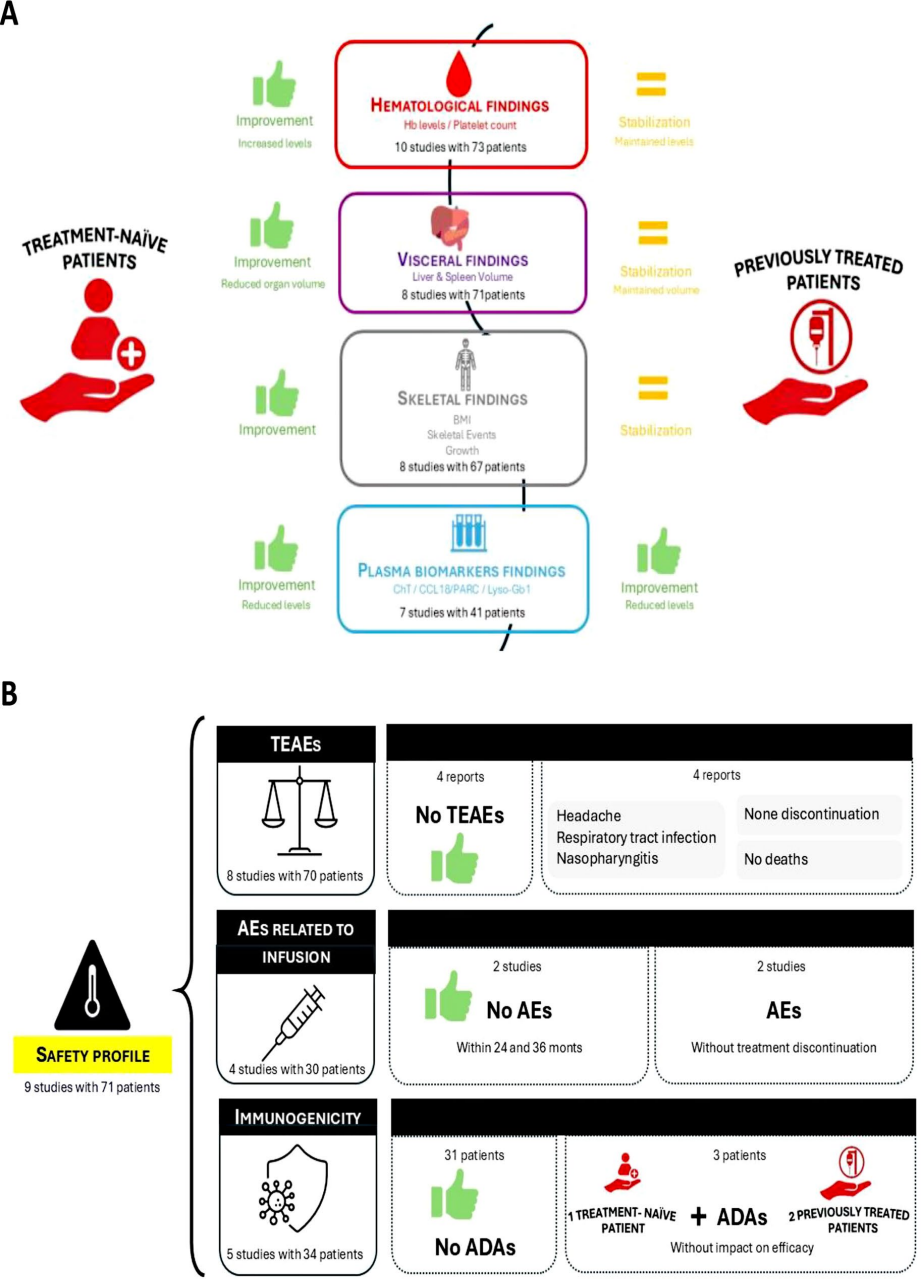
Key efficacy/effectiveness (**A**) and safety (**B**) outcomes for paediatric patients with GD1 across all age groups. ADA: Anti-velaglucerase alfa antibody; AE: Adverse event; BMI: Bone marrow infiltration; CCL18/PARC: Chemokine (C–C motif) ligand 18 (CCL18)/pulmonary and activation-regulated chemokine (PARC); ChT: Chitotriosidase; Hb: Haemoglobin; Lyso-Gb1: Glucosylsphingosine; TEAE: Treatment emergent adverse event

Out of 23 publications, 9 (39.13%, *n* = 71 patients) incorporated data on the safety parameters of velaglucerase alfa in paediatric patients with GD1 (Table 3) [32–35, 37, 43, 44, 47]. The primary findings regarding safety outcomes are presented in Fig. 2B and have been provided in advance.

**Table 3:** Patient safety observations. Type 1 Gaucher disease

Author, year [reference]	Treatment status (number of patients)	Adverse events		Immunogenicity
		Treatment emergent	Others	
Becker-Cohen, 2023 [26]	N (1)	There were patients with TEAEs, but it was not specified whether the paediatric patient was one of them.	No severe AEs were associated with infusion acceleration.	ND
Ida, 2016 [32]	PTP (1)	None of the AEs were considered TEAEs.	The patient experienced severe AEs	The patient tested negative for ADAs.
Pastores, 2016 [33]	PTP (1)	No TEAEs were reported.	The patient received acetaminophen during the trial extension as premedication to prevent possible infusion reactions. The patient experienced at least one AE during treatment but did not discontinue the trial due to this AE.	The patient tested positive for ADAs. The ADAs were neutralizing activity positive. This patient had been previously treated with imiglucerase, testing positive for anti-imiglucerase antibodies. The presence of neutralizing ADAs did not impact the evaluated efficacy parameters.
Smith, 2016 [34]	N (10) and PTP (14)	The most common TEAEs were respiratory tract infection, nasopharyngitis, and headache. Seven patients experienced at least 1 TEAE. Three patients experienced at least 1 AE related to treatment infusion. Only 1 AE, calf muscle pain, was related to velaglucerase alfa use.	All patients reported at least one AE during the extension study. Eight patients experienced 13 bone-related AEs. No deaths or discontinuations were observed due to AEs. One patient experienced 3 serious AEs, respiratory tract infection, bronchopneumonia, and a seizure, none of which were related to treatment.	In total, 7.14% (1/14) of the PTP patients tested positive for ADAs that were neutralizing activity positive. Those patients had been previously treated with imiglucerase, testing positive for anti-imiglucerase antibodies. The presence of neutralizing ADAs did not impact the evaluated efficacy parameters.
Pastores, 2015 [43]	PTP (1)	ND		The patient tested positive for ADAs. The ADAs were neutralizing activity positive. The relationship between the presence of neutralizing ADAs and the lack of or improvement in efficacy parameters was not reported.
Pastores, 2014 [44]	N (10) and PTP (14)	Seven patients experienced at least one TEAE.	One patient experienced 3 serious AEs, none of which were related to treatment. No deaths or discontinuations were observed due to AEs.	ND
Turkia, 2013 [35]	N (4)	The most common AEs were related to treatment infusion. There were serious emergent AEs during treatment.	No deaths or discontinuations were observed due to AEs.	ND
Zimran, 2013 [37]	PTP (9)	None of the reported AEs were associated with treatment.	There were no life-threatening AEs.	ND
Zimran, 2010 [47]	ND (7)	No paediatric patients experienced serious emergent AEs during treatment.	No deaths or discontinuations were observed due to AEs.	No patient tested positive for ADAs.

ADA: Anti-velaglucerase alfa antibody; AE: Adverse event; GD: Gaucher disease; N: Treatment-naïve; ND: No data; PTP: Patient previously treated with imiglucerase; TEAE: Treatment emergent adverse event

#### TEAEs

TEAEs were reported in 8 out of 23 articles (34.78%, *n* = 70 patients). No TEAEs were observed in 4 reports that included patients with GD1 [32, 33, 37, 47]. Conversely, 4 publications noted patients experiencing TEAEs, with the most common being headache, respiratory tract infection and nasopharyngitis. Nevertheless, no deaths were reported, and none of the patients discontinued the treatment due to these TEAEs [26, 34, 35, 44].

Two studies reported no adverse events (AEs) related to treatment infusion within 24 and 36 weeks of follow-up [26, 33]; another two studies reported AEs related to the infusion of velaglucerase alfa, but none of the affected patients had to stop treatment [34, 35].

#### Immunogenicity

Immunogenicity was described in 5 out of 23 publications (21.74%, *n* = 34 patients), in which most of the included patients (91.18%, *n* = 31 patients) did not develop ADAs [32–34, 43, 47]. However, a total of 3 patients (8.82%) tested positive for neutralizing ADAs [33, 34, 43]. Nevertheless, two articles noted that the presence of antibodies did not appear to impact the efficacy of the treatment [33, 34]. Among the 3 patients with neutralizing ADAs, one was treatment-naïve, whereas the other 2 patients were previously treated with imiglucerase, to which they had developed anti-imiglucerase antibodies [33, 34, 43].

### Efficacy/effectiveness profile of velaglucerase alfa in the treatment of paediatric patients with GD1

Overall, 14 out of 23 publications (60.87%, *n* = 113 patients) involving paediatric patients with GD1 reported the effects of velaglucerase alfa on at least one of the evaluated efficacy/effectiveness parameters (Table 4) [26, 27, 31–35, 37, 40, 42, 44–47]. The main results related to efficacy/effectiveness outcomes are displayed in Fig. 2A and have been made available beforehand.

**Table 4:** Patient efficacy/effectiveness observations. Type 1 Gaucher disease

Author, year [reference]	Treatment status (number of patients)	Haematological parameters	Visceral parameters	Skeletal parameters	Plasma biomarkers	Quality of life
Becker-Cohen, 2023 [26]	N (1)	The Hb concentration increased from 11.6 to 14.5 g/dL. The platelet count improved from 77 to 95 × 10^6^/mL.	The volume of the liver decreased from 2.54 to 1.69 MN. The volume of the spleen reduced from 22.23 to 12.75 MN.	ND	No data on ChT activity or CCL18/PARC concentration were reported. The concentration of Lyso-Gb1 reduced from 470 to 276 ng/mL.	The quality of life was assessed but was not disaggregated by patient age.
Stiles, 2021 [27]	N (1)	The Hb concentration and platelet count were within normal ranges prior to the initiation of the treatment.	ND	The BMD was within normal limits prior to the initiation of treatment (Z score = 1.6 for the spinal cord and 1.9 for the whole body). No skeletal events were reported. Height, weight, and head circumference were appropriate for age.	No data on ChT activity or CCL18/PARC concentration were reported. The concentration of Lyso-Gb1 was elevated before treatment onset (45.8 ng/L). There was a marked reduction of these levels at 3 months of treatment.	ND
Zimran, 2018 [31]	N (8)	ND	ND	No skeletal events were reported. All paediatric patients (7/7) reached the 5th height percentile (the study’s goal).	ND	ND
Elstein, 2016 [40]	N (15)	ND	ND	No skeletal events were reported. Three patients below the 5th height percentile achieved the therapeutic goal of reaching the 5th percentile at 3 y. One of two patients reached it at 4 y.	ND	ND
Ida, 2016 [32]	PTP (1)	The haematological parameters remained stable. The Hb concentration was 14.1 g/dL. The platelet count was 231 × 10^9^/L.	The volumes of the liver and spleen remained stable throughout the observation period. The volume of the liver was 0.90 MN. The volume of the spleen was 2.12 MN.	The total BMB score decreased by 4 points. No skeletal events were reported. The height Z score changed by 0.4, placing the patient at the 24.4th percentile at 24 months	The ChT activity was not assessed due to the patient status as homozygous (dup24/dup24) for a 24-bp insertion in exon 10 of the *CHIT1* gene (NM_003465.3:c.1049_1072dup24) [73]. No data on CCL18/PARC or Lyso-Gb1 concentration were reported.	ND
Pastores, 2016 [33]	PTP (1)	The haematological parameters remained stable after treatment upgrade.	ND	ND	The ChT activity and CCL18/PARC concentration remained stable after treatment upgrade. No data on Lyso-Gb1 concentration were reported.	ND
Smith, 2016 [34]	N (10) and PTP (14)	The Hb concentration (changed by 2.65 g/dL, 24.3%) and platelet count (changed by 79.13 × 10^9^/L, 93.4%) of the N patients increased. These parameters remained stable in PTP patients: the Hb concentration changed by 0.13 g/dL (1.2%), and platelet count changed by 18.22 × 10^9^/L (8.6%).	The volumes of the liver and the spleen reduced in N patients (the volume of the liver changed by -1.14 MN (-27.5%), and the volume of the spleen changed by -2.55 MN (-66.3%)). These parameters remained stable in PTP patients throughout the observation period: the volume of the liver changed by -0.11 MN (-5.0%), and the volume of the spleen changed by 0.04 MN (-4.1%).	The total BMB score decreased in both groups of patients (changed by -3.2 in N patients and − 0.1 in PTP patients). One patient reported bone pain; treatment status (N or PTP) was not declared by the authors. The height Z score increased in N patients (0.595) and was stable in PTP patients (0.068).	The ChT activity and CCL18/PARC concentration decreased in N patients (changed by -35,132 nmol/mL/h (91.5%) and − 1720 ng/mL (-91.2%), respectively). The ChT activity and CCL18/PARC concentration decreased in PTP patients (changed by -1607 nmol/mL/h (-24.0%) and − 191 ng/mL (52.6%), respectively). No data on Lyso-Gb1 concentration were reported in either N or PTP patients.	ND
Bailey, 2015 [42]	PTP (1)^1^	The haematological parameters were stable at treatment onset. The haematological parameters remained stable throughout the observation period.	At baseline, the volume of the liver was within the normal range, while the volume of the spleen was slightly enlarged. The volumes of the liver and the spleen remained stable throughout the observation period.	No bone pain or crises were reported throughout the observation period.	ND	The quality-of-life score was improved from 1 to 8 out of 10.
Pastores, 2014 [44]	N (10) and PTP (14)	The Hb concentration and platelet count remained stable in N patients and exhibited further improvement in PTP patients.	The volumes of the liver and spleen remained stable in N patients and showed further improvement in PTP patients.	ND	ND	ND
Giraldo, 2013 [45]	PTP (9)	ND	ND	The bone marrow infiltration remained stable after treatment upgrade. The height scores remained stable after treatment upgrade.	ND	ND
Turkia, 2013 [35]	N (4)	The Hb concentration increased to 12.5 g/dL and the platelet count increased to 1.3–1.7 times the initial count in all patients.	The volume of the liver decreased by 18.5% to 29.4%. The volume of the spleen decreased by 36.2% to 61.3%.	ND	The ChT activity (changed from − 19,864 to -37,229 nmol/mL/h) and CCL18/PARC concentration (changed from − 372 to -1916 ng/mL) decreased. No data on Lyso-Gb1 concentration were reported.	ND
Zimran, 2013 [46]	N (8)	ND	ND	The BMB of the spinal cord and lumbar decreased by ≥ 2 points at 5 y. The height Z score changed by 1.21.	ND	ND
Zimran, 2013 [37]	PTP (9)	A total of 66.7% (6/9) of patients experienced improvements in all haematological parameters. The Hb concentration increased to 12.2 g/dL and the platelet count increased to between 153 and 369 × 10^9^/L in all patients.	The volume of the liver increased in all patients (by 4.2% to 19.6%). The volume of the spleen increased in 55.5% (5/9) of patients (by 1.7% to 29.0%), remained stable in 11.1% (1/9) of patients, and decreased in 33.3% (3/9) of patients (by 1.6% to 6.9%)	ND	The ChT activity and CCL18/PARC concentration decreased in 88.9% (8/9) of patients. No data on Lyso-Gb1 concentration were reported.	ND
Zimran, 2010 [47]	ND (7)	The Hb concentration increased by 16.3% to 24.7%. The platelet count increased by 26.4% to 38.9%.	The volume of the liver decreased by 10.6% to 18.3%. The volume of the spleen decreased by 47.6% to 64.6%.	ND	ND	ND

BMB: Bone marrow burden; BMD: Bone mineral density; CCL18/PARC: Chemokine (C–C motif) ligand 18/pulmonary and activation-regulated chemokine; ChT: Chitotriosidase; GD: Gaucher disease; Hb: Haemoglobin; Lyso-Gb1: Glucosylsphingosine; MN: Multiples of normal; N: Treatment-naïve; ND: No data; PTP: Patient previously treated with imiglucerase

^1^ Patient treated with imiglucerase and then treated with velaglucerase alfa alone and then in combination with eliglustat

#### Haematological findings

Out of 23 studies, 10 (43.48%, *n* = 73 patients) included data on the Hb concentration and/or platelet count, demonstrating that treatment with velaglucerase alfa either maintained or improved these parameters [26, 27, 32–35, 37, 42, 44, 47]. Most studies indicated that treatment-naïve patients experienced improvements in haematological parameters [26, 34, 35, 44], whereas these parameters remain stable in patients previously treated with imiglucerase [32–34, 44]. Nevertheless, two studies reported two patients whose haematological variables were normal prior to initiating treatment with velaglucerase alfa or a combination of velaglucerase alfa and eliglustat and whose levels remained stable after treatment initiation [27, 42]. Additionally, improvements in these factors were observed in patients previously treated with imiglucerase [37], as well as in patients with unknown treatment status [47].

#### Visceral findings

Information regarding visceral parameters was reported in 8 out of 23 studies (34.78%, *n* = 71 patients). Five of these articles reported that treatment with velaglucerase alfa resulted in a reduction in the volumes of the liver and the spleen in naïve patients and the stabilization of these factors in patients previously treated with imiglucerase [26, 32, 34, 35, 44]. However, the volumes of both organs remained stable in a patient with slightly enlarged volumes and who was treated initially with a combination of velaglucerase alfa and eliglustat [42]. Additionally, one study reported an increase after 12 months of treatment in the volume of the liver in 9 patients and in the volume of the spleen in 5 patients, while these volumes were maintained in one patient and decreased in 3 patients previously treated with imiglucerase [37]. Nonetheless, a study that did not provide details about the treatment status of the included patients reported a reduction in the volumes of both organs following treatment with velaglucerase alfa [47].

#### Skeletal findings

Data regarding the effects of velaglucerase alfa on bone parameters were recorded in 8 out of 23 publications (34.78%, *n* = 67 patients).

##### Bone mineral density

The effect on BMD was reported only in one treatment-naïve patient. This patient’s Z score was within the normal range prior to treatment and remained within the normal range following the initiation of velaglucerase alfa therapy [27].

##### Bone marrow infiltration

Bone marrow infiltration, assessed using bone marrow burden (BMB) scores, was included in 4 out of 23 studies (17.39%, *n* = 42 patients). One publication involving 8 treatment-naïve patients demonstrated that velaglucerase alfa treatment resulted in improved BMB scores [46]. Another report indicated that BMB scores improved following velaglucerase alfa treatment in naïve patients [34]. In patients previously treated with imiglucerase, BMB scores generally remained stable after switching to velaglucerase alfa [34, 45]. However, lower BMB scores were recorded for 2 patients previously treated with imiglucerase, whereas an increased BMB score was mentioned for another previously treated patient [32].

##### Skeletal events

Bone crises and pain were indicated in 3 out of 23 records (13.04%, *n* = 26 patients). Overall, only one out of 25 patients (4.0%) with GD1 experienced bone pain [27, 34]. Additionally, a patient who underwent combination therapy (velaglucerase alfa with eliglustat) did not report any bone pain or crises [42]. None of the studies analysed described bone vascular phenomena leading to infarctions or osteonecrosis.

##### Growth (weight and height)

Information regarding height was noted in 7 out of 23 publications (30.43%, *n* = 66 patients). Patients treated with velaglucerase alfa, whether treatment-naïve or previously treated with imiglucerase, exhibited an improvement or stabilization in the height Z score and height percentiles [27, 31, 32, 34, 40, 45, 46]. No data related to weight scores and/or percentiles were retrieved.

#### Plasma biomarker findings

The effect of treatment on plasma biomarkers was described in 7 out of 23 publications (30.43%, *n* = 41 patients). The concentrations of the three biomarkers assessed in the present review were reduced following treatment with velaglucerase alfa. The ChT activity and the concentration of CCL18/PARC were reduced in the 43 patients included in 4 of these publications [33–35, 37]. Moreover, a reduction in the concentration of Lyso-Gb1 was recorded in two patients [26, 27].

#### Health-related quality of life findings

The impact of velaglucerase alfa treatment on the QoL of these patients was reported in 2 out of 23 publications (8.69%, *n* = 10 patients). A case study described an improvement in a patient’s QoL score (tool not disclosed by the authors) from 1 to 8 out of 10 after combined treatment with velaglucerase alfa and eliglustat [42]. Another study that included 9 paediatric patients reported improvements in patient-reported outcomes [35].

Additionally, during this review, 2 studies (8.69%, *n* = 2) reported that the use of velaglucerase alfa in a home setting could be considered effective and safe for the treatment of paediatric patients with GD1 [26, 42].

### Velaglucerase alfa in the treatment of paediatric patients with GD3

The safety profile of velaglucerase alfa treatment in paediatric patients with GD3 was described in 7 out of 23 publications (30.43%, *n* = 26 patients) (Table 5) [29, 30, 32, 33, 36, 41, 43]. Information on TEAEs was included in 5 articles (21.74%, *n* = 24 patients). No TEAEs were reported in 2 of the studies [32, 33]. However, 3 patients experienced AEs potentially related to treatment [29, 30, 41]. Immunogenicity data were provided in 6 publications (26.09%, *n* = 17 patients) [30, 32, 33, 36, 41, 43]. In total, 4 patients (23.53%) tested positive for ADAs. The neutralizing activity of these antibodies was reported in 1 patient (5.88%) [33], non-neutralizing activity was reported in another patient (5,88%) [43], and the nature of the activity was not specified in the remaining two patients (11.76%) [30, 41]. For one of these latter patients, the presence of antibodies did not appear to influence the efficacy of the treatment [41].

**Table 5:** Patient safety observations. Type 3 Gaucher disease

Author, year [reference]	Treatment status (number of patients)	Adverse events		Immunogenicity
		Treatment emergent	Others	
Schwartz, 2018 [29]	N (5) and PTP (4)	A 3-y-old girl (1/9, 10.1%) reported 3 AEs (stare, cold and cough) that could be related to treatment. The treatment status of the girl (N or PTP) was not declared by the authors.	ND	ND
Tantawy, 2018 [30]	N (6)	The only TEAE was headache in 1 patient.	All patients reported AEs.	A total of 16.7% (1/6) of patients tested positive for ADAs, but antibody levels decreased over time. The ADAs were neutralizing activity negative. The relationship between the presence of no neutralizing ADAs and the lack of or improvement in efficacy parameters was not reported.
Ida, 2016 [32]	PTP (2)	None of the reported AEs were associated with treatment.	A total of 50.0% (1/2) of patients experienced severe AEs.	None of the patients tested positive for ADAs.
Pastores, 2016 [33]	N (1)	No infusion-related AEs were reported. The patient experienced at least one emergent AE during treatment.	The patient did not discontinue the trial due to AEs.	The patient tested positive for ADAs. The ADAs were neutralizing activity negative. The presence of non-neutralizing ADAs did not impact the improvement of the evaluated efficacy parameters.
Tantawy, 2016 [41]	N (6)	All patients reported emergent AEs during treatment. The only AE due to treatment was headache in 1 patient.	ND	A total of 16.7% (1/6) of patients tested positive for ADAs. The ADAs were neutralizing activity negative. The presence of non-neutralizing ADAs did not impact the evaluated efficacy parameters.
Pastores, 2015 [43]	N (1)	ND		The patient tested positive for ADAs. The non-neutralizing ADAs were neutralizing activity negative. The relationship between the presence of ADAs and the lack of or improvement in efficacy parameters was not reported.
Vairo, 2013 [36]	PTP (1)	ND		The patient tested negative for ADAs.

ADA: Anti-velaglucerase alfa antibody; AE: Adverse event; GD: Gaucher disease; N: Treatment-naïve; ND: No data; PTP: Patient previously treated with imiglucerase; TEAE: Treatment-emergent adverse event

The effectiveness of velaglucerase alfa in the treatment of paediatric patients with GD3 was noted in 5 out of 23 studies (21.74%, *n* = 16 patients) (Table 6) [30, 32, 33, 36, 41]. All 5 publications included information on haematological factors. Treatment with velaglucerase alfa resulted in increased the Hb concentrations and platelet counts in treatment-naïve patients [30, 33, 41], and these parameters were either maintained or increased in patients previously treated with imiglucerase [32, 36]. Visceral parameters were observed in 4 out of 23 articles (17.39%, *n* = 15 patients). A reduction in the volumes of the liver and the spleen was registered in treatment-naïve patients [30, 41], whereas one study described stable volumes in patients previously treated with imiglucerase [32]. Another manuscript described a patient with GD3 previously treated with imiglucerase who experienced a decrease in the volume of the spleen but an increase in the volume of the liver [36]. Regarding skeletal variables, 2 out of 23 records (8.69%, *n* = 8 patients) described the effect of velaglucerase alfa. One article reported a decrease in the BMB score in one patient previously treated with imiglucerase, whereas another patient experienced an increase in this score [32]. In terms of the height Z score, one treatment-naïve patient remained stable, and 4 did not reach the normal height for their age group [41]. Additionally, two patients previously treated were at a lower height percentile despite velaglucerase alfa treatment [32]. Plasma biomarkers were communicated in 4 out of 23 studies (17.39%, *n* = 10 patients). The ChT activity and the concentration of CCL18/PARC were reduced in treatment-naïve patients [30, 33, 36], although Lyso-Gb1 concentration was not reported. Finally, a case report of one patient showed an improvement in QoL, as evaluated by the SF-36, which was completed by the patient’s caregiver [36].

**Table 6:** Patient effectiveness observations. Type 3 Gaucher disease

Author, year [reference]	Treatment status (number of patients)	Haematological parameters	Visceral parameters	Bone parameters	Plasma biomarkers	Quality of life
Tantawy, 2018 [30]	N (6)	The Hb concentration increased in all patients (mean 2.2 g/dL), and 83.3% (5/6) of patients reached normal values. The platelet count increased in 83.3% (5/6) of patients (mean 136.6 × 10^9^/L).	The volumes of the liver and the spleen decreased in all patients with data (means: -62.3 and − 30.1 MN, respectively)	ND	The ChT activity and CCL18/PARC concentration were reduced in 80.0% (4/5) of patients with data (means: -58.0 nmol/mL/h and − 58.2 ng/mL, respectively). The concentrations of both biomarkers were within normal ranges at baseline. No data on Lyso-Gb1 concentration were reported.	ND
Ida, 2016 [32]	PTP (2)	The haematological parameters remained stable. The Hb concentration was above 14 g/dL. The platelet count was above 150 × 10^9^/L.	The volumes of the liver and the spleen remained stable. The volume of the liver was below 0.76 MN. The volume of the spleen was below 2.72 MN.	The total BMB score decreased in one patient but increased in the other. Both patients were at a lower height percentile.	The ChT activity was not assessed due to due to the status of both patients as homozygous (dup24/dup24) for a 24-bp insertion in exon 10 of the *CHIT1* gene (NM_003465.3:c.1049_1072dup24). No data on CCL18/PARC or Lyso-Gb1 concentration were reported.	ND
Pastores, 2016 [33]	N (1)	The Hb concentration and platelet count were improved.	ND	ND	The ChT activity and CCL18/PARC concentration were improved. No data on Lyso-Gb1 concentration were reported	ND
Tantawy, 2016 [41]	N (6)	The Hb concentration increased from 9.6 to 11.8 g/dL. The platelet count increased from 86.0 × 10^9^/L to 220.8 × 10^9^/L.	The volumes of the liver and the spleen decreased from 1.8 to 1.3 MN and from 20.3 to 6.5 MN, respectively.	A total of 80.0% (4/5) of patients who were below the therapeutic goal of reaching the 5th height percentile at the beginning of the study remained below this level after 12 months of treatment.	ND	ND
Vairo, 2013 [36]	PTP (1)	The Hb concentration increased from 8.0 to 12.6 g/dL. The platelet count increased from 56 × 10^3^/mm^3^ to 115 × 10^3^/mm^3^.	The volume of the liver was considered to remain stable, increasing from 5367 to 5369 cm^3^. The size of the spleen decreased from 27 to 17 cm.	ND	The ChT activity decreased from 19,878 to 13,074 nmol/mL/h. No data on CCL18/PARC and Lyso-Gb1 concentration were reported.	The quality-of-life was improved (SF-36 filled out by the patients’ caregivers).

BMB: Bone marrow burden; CCL18/PARC: Chemokine (C–C motif) ligand 18/pulmonary and activation-regulated chemokine; ChT: Chitotriosidase; Hb: Haemoglobin; GD: Gaucher disease; MN: Multiples of normal; N: Treatment-naïve; ND: No data; PTP: Patient previously treated with imiglucerase

## Discussion

A systematic review of the literature was conducted primarily to aggregate the existing evidence pertaining to the safety and efficacy/effectiveness of velaglucerase alfa administration in patients with GD1 across all paediatric ages (0–18 y). Concurrently, the review also aimed to gather the available data on the safety and efficacy/effectiveness of velaglucerase alfa treatment in paediatric patients with GD3.

To the best of the authors’ knowledge, this constitutes the first systematic review investigating the administration of velaglucerase alfa in the paediatric population of patients with GD. A recent publication presented a systematic review and meta-analyses of GD [48]. However, all patients with GD, irrespective of their age and type of GD, were included, and all available therapeutic options, including both ERT and SRT approaches, were considered. Therefore, to the authors’ understanding, this work is important because it describes the safety and efficacy/effectiveness of velaglucerase alfa in the treatment of paediatric patients with GD, given that early intervention influences the benefits accrued [5, 18].

The level of evidence of the publications recorded in this review included 13 clinical trials [26, 30–32, 34, 35, 37, 40, 41, 44–47], 5 observational studies [25, 29, 33, 38, 43] and 5 case studies [27, 28, 36, 39, 42]. Despite the substantial representation of clinical trials in this sample, these studies typically involved small sample sizes, a characteristic/limitation shared by research in rare diseases, even more so if it is exclusively targeted at the paediatric population [49, 50]. Therefore, the impact of velaglucerase alfa on certain parameters has been reported only for a limited number of patients. For example, the effect of velaglucerase alfa on BMD scores has been documented in a single patient with GD1. Similarly, while information on HRQoL was available for 21 paediatric patients, data could be extracted from only two of them [21, 26]. During the full-text screening process, numerous publications that included our target population (paediatric patients with GD1 or GD3) had to be excluded, as the results were not disaggregated by age, preventing the extraction of data specific to paediatric patients. This underscores the need for further research in this field to amass the requisite knowledge on the disease and its treatment with velaglucerase alfa in paediatric patients.

The administration of velaglucerase alfa as a treatment for paediatric patients with GD1 may be a beneficial option in both treatment-naïve and previously treated patients with imiglucerase. None of the TEAEs recorded in these patients led to death or discontinuation of treatment [26, 32–35, 37, 44, 47]. Additionally, the evidence collected during this review indicates that the formation of ADAs does not seem to affect the efficacy of this treatment [33, 34].

During this analysis, data were obtained for all efficacy parameters under study. Overall, the evidence collected during the systematic review may suggest that these therapeutic goals could potentially be achieved with the administration of velaglucerase alfa, as inidicated by the observed improvement in these parameters among treatment-naïve patients and the maintenance of parameters in patients previously treated with imiglucerase. In addition, our research gathered variables related to the efficacy of ERT treatment, which were not currently considered therapeutic objectives [7, 20]. These parameters included the plasma concentrations of CCL18/PARC and Lyso-Gb1 as well as ChT activity. The analysis revealed a general reduction in the levels of these three biomarkers under study [26, 27, 33–35, 37]. Although some experts believe that fluctuations in ChT activity and CCL18/PARC and Lyso-Gb1 concentrations should not be considered therapeutic goals for treatment, as previously mentioned [7, 20], these measurements could serve as valuable tools for monitoring disease progression and evaluating the therapeutic response in patients with GD, potentially leading to treatment optimization [7, 51]. In fact, one study showed a correlation between Lyso-Gb1 concentration and disease severity, noting that a significant increase in this concentration is associated with a more severe GD phenotype [52]. Furthermore, a meta-analysis provided evidence supporting the association of the ChT activity and the concentration of CCL18/PARC with abnormalities in haematological and visceral manifestations in both treated and untreated patients [53]. In this context, establishing recommendations and objectives for the interpretation of biomarkers in relation to the personalization of patient treatment becomes necessary.

The progression of the disease and/or the response to treatment can significantly influence HRQoL [54]. Consequently, enhancing QoL scores is also viewed as a management objective for patients with GD across all ages (paediatric and adult patients). Throughout this review, different studies indicate an improvement in the QoL of paediatric patients with GD1, as evidenced by increased scores in various questionnaires [35, 42, 55]. However, the number of publications reporting HRQoL insights in paediatric patients with GD is limited [55], despite the availability of various self-report questionnaires and patient-reported outcome measures (PROMs) to assess HRQoL in these patients [56]. Indeed, various PROMs have proven useful for assessing lysosomal storage disorders [57], but there are currently no PROMs specifically developed or validated for paediatric patients with GD and/or their caregivers [58]. Furthermore, specific PROMs have been developed to measure HRQoL in children [59] and could therefore be beneficial in assessing the effects of specific treatments (ERT and/or SRT) on the QoL of this patient population. The use of PROMs in clinical practice can foster patient-centred care and enhance QoL, as well as symptom management [54]. The administration of treatment via home infusion has been identified as a factor contributing to improving the QoL in patients with GD, as lifelong hospital administration can have a negative impact [60]. Additional benefits, such as flexible administration times, allow for the adjustment of treatment infusion to the daily routine of patients and increased treatment compliance [61, 62]. This review identified several studies that describe the potential safety and feasibility of home administration of velaglucerase alfa in patients with GD of all paediatric ages [26, 36, 42]. Although the number of publications specifically describing home infusion in this population is limited and the sample size is small, the results align with those reported in previous studies that included both paediatric and adult patients with GD [63–65]. Overall, all the publications concluded that the home use of velaglucerase alfa could be considered safe and enhance treatment compliance. Furthermore, it has been reported that all patients with GD who received home infusions would choose home administration again if given the option, as they found it helped them better manage their disease and perceived the quality of care to be equivalent to that received in a hospital setting [66]. The implementation of home therapy has also been shown to ensure the continuity of treatment during times of social disruption (e.g., the COVID-19 pandemic), among others [67]. Currently, there is a scarcity of published data, despite a higher number of patients with GD receiving home therapy than records indicate. A call to action is desirable to encourage the publication of patients’ experiences with the home treatment model.

With respect to velaglucerase effectiveness in paediatric patients with GD3, the practice guidelines support that although ERT has not been proven to be effective in treating nGD, it could be a treatment option for non-neurological manifestations [68].

The data recorded during our study suggest that velaglucerase alfa may be considered a safe treatment for patients with GD3, with no deaths or treatment discontinuations due to TEAEs [29, 30, 32, 33, 41]. Additionally, the presence of ADAs did not appear to interfere with the efficacy of velaglucerase alfa in managing the non-neurological manifestations of the disease [41]. This aligns with the findings reported in prior studies [68].

Indeed, although limited, the evidence documented in this review suggests that velaglucerase alfa treatment may improve haematological and visceral symptoms in all ages of paediatric patients with GD3 [30, 32, 33, 36, 41]. Additionally, this systematic review describes a reduction in the concentration of plasma biomarkers and an improvement in the QoL among patients with GD3 of all paediatric ages. This may represent an important observation, as it suggests that velaglucerase alfa treatment not only addresses the physical symptoms of the disease but also enhances the overall well-being of patients [30, 33, 36]. These findings should be interpreted with caution because the sample size was small, and no specific controlled trials have yet been established for this subpopulation. Nonetheless, they align with observations from the long-term use of velaglucerase alfa, under the physician criteria, in both paediatric and adult patients [69], and with the results described with the use of imiglucerase in these patients [70]. These observations suggest the potential safety and effectiveness of long-term use of these treatments. Therefore, the results of this systematic review indicate possible benefits of long-term treatment with velaglucerase alfa for non-neurological symptoms in patients with GD3. Although established ERT molecules do not cross the BBB, some patients do not deteriorate neurologically when treated with ERT [1]. This effect might be explained by a reduction in systemic neuroinflammation, which is associated with a reduced microglial response to cytokines and chemokines, as well as a reduction in the burden of Gaucher cells [70–72]. However, further studies should explore the effects of velaglucerase alfa on the non-neurological and neurological manifestations of paediatric patients with GD3.

In addition, different limitations should be considered when interpreting the findings of this review. First, as already mentioned, availability of evidence was scarce and the overall sample size was small, which limits the generalizability of the results. Second, due to the descriptive nature of these studies, a formal risk of bias assessment was not performed. Third, the reporting and categorization of AEs were limited by the nature of the included studies, and the way in which AEs were reported, with most of the studies describing aggregated safety data. As a result, systematic grouping was not feasible. Finally, although the review protocol was developed in advance and followed rigorously, it was not registered in a public database such as PROSPERO, which is recommended. These factors should be considered when interpreting the conclusions of this review. Nevertheless, these limitations do not diminish the value of this study, which provides relevant information by compiling the available evidence and may support clinical decision-making in this context, while underscoring the need for further research.

To increase the level of evidence supporting the use of velaglucerase alfa in patients with GD (GD1 or GD3) across all paediatric ages (0–18 y), it would be essential to conduct targeted studies for this specific population. These studies should incorporate the primary parameters used to evaluate this disease. Moreover, it would be beneficial to include additional variables such as the HRQoL of caregivers. This is a parameter for which no data have been collected thus far, yet it is of significant interest considering the burden associated with caring for a paediatric patient with this disease. The inclusion of such factors could provide a more comprehensive understanding of the impact of the disease and its treatment, not only for patients but also for those who care for them. This holistic approach could lead to more effective and patient-centred treatment strategies for GD.

## Conclusions

This systematic literature review suggests that velaglucerase alfa may represent a benefitial treatment option for paediatric patients of all ages with GD1. It reports observed improvements in haematological, visceral, and skeletal parameters, as well as biomarker levels and quality of life, in both treatment-naïve patients and patients previously treated with imiglucerase. Although there is limited published evidence regarding the use of velaglucerase alfa in patients with GD3, the studies included in this analysis suggests that velaglucerase alfa appears to be beneficial for treating non-neurological manifestations in paediatric patients with GD3 of all ages (0–18 y). While additional studies with larger populations of paediatric patients with GD are needed, these findings highlight the potential of velaglucerase alfa as an effective treatment for non-neurological symptoms in paediatric patients of all ages with GD1 or GD3. Further research is needed to confirm these results and assess their impact on other disease aspects.

## Electronic supplementary material

Below is the link to the electronic supplementary material.

Supplementary Material 1

## Data Availability

All data generated or analysed during this study are included in this published article and its supplementary information files.

## Abbreviations

ADAs: Anti-velaglucerase alfa antibodies
AE: Adverse event
BBB: Blood-Brain-Barrier
BMB: Bone marrow burden
BMD: Bone mineral density
CCL18/PARC: Chemokine (C–C motif) ligand 18 (CCL18)/pulmonary and activation-regulated chemokine (PARC)
ChT: Chitotriosidase
CNS: Central nervous system
ERT: Enzyme replacement therapy
GCase: β-glucosidase/glucocerebrosidase
GD: Gaucher disease
GD1: Type 1 Gaucher disease
GD2: Type 2 Gaucher disease
GD3: Type 3 Gaucher disease
GluCer: Glucosylceramide
Hb: Haemoglobin
HRQoL: Health-related quality of life
ICIEM: International Congress of Inborn Errors of Metabolism
Lyso-Gb1: Glucosylsphingosine
nGD: Neurological Gaucher disease
PedsQL: Pediatric Quality of Life Inventory
PICO: Population, interventions, comparisons, outcomes
PRISMA: Preferred Reporting Items for Systematic Reviews and Meta-Analyses
PROM: Patient-reported outcome measures
QoL: Quality of life
SF-36: The 36-Item Short Form Health Survey
SIMD: Society for Inherited Metabolic Disorders
SRT: Substrate reduction therapy
SSIEM: Society for the Study of Inborn Errors of Metabolism
TEAEs: Treatment emergent adverse events

## References

[1] Castillon G, Chang S-C, Moride Y. Global incidence and prevalence of Gaucher disease: A targeted literature review. JCM. 2022;12:85. 36614898 10.3390/jcm12010085 PMC9821068

[2] Stirnemann J, Belmatoug N, Camou F, Serratrice C, Froissart R, Caillaud C, et al. A review of Gaucher disease Pathophysiology, clinical presentation and treatments. IJMS. 2017;18:441. 28218669 10.3390/ijms18020441 PMC5343975

[3] Furderer ML, Hertz E, Lopez GJ, Sidransky E. Neuropathological features of Gaucher disease and Gaucher disease with parkinsonism. IJMS. 2022;23:5842. 35628652 10.3390/ijms23105842 PMC9147326

[4] Gary SE, Ryan E, Steward AM, Sidransky E. Recent advances in the diagnosis and management of Gaucher disease. Expert Rev Endocrinol Metabolism. 2018;13:107–18. 10.1080/17446651.2018.1445524 PMC6129380 30058864

[5] Weinreb NJ, Goker-Alpan O, Kishnani PS, Longo N, Burrow TA, Bernat JA, et al. The diagnosis and management of Gaucher disease in pediatric patients: where do we go from here? Mol Genet Metab. 2022;136:4–21. 35367141 10.1016/j.ymgme.2022.03.001

[6] Goker-Alpan O, Ivanova MM. Neuronopathic Gaucher disease: rare in the West, common in the East. J Inher Metab Disea. 2024;47:917–934. 10.1002/jimd.12749 38768609

[7] Biegstraaten M, Cox TM, Belmatoug N, Berger MG, Collin-Histed T, Vom Dahl S, et al. Management goals for type 1 Gaucher disease: an expert consensus document from the European working group on Gaucher disease. Blood Cells Molecules Dis. 2018;68:203–8. 10.1016/j.bcmd.2016.10.008 28274788

[8] Kong W, Lu C, Ding Y, Meng Y. Update of treatment for Gaucher disease. Eur J Pharmacol. 2022;926:175023. 35569551 10.1016/j.ejphar.2022.175023

[9] Larsen SD, Wilson MW, Abe A, Shu L, George CH, Kirchhoff P, et al. Property-based design of a glucosylceramide synthase inhibitor that reduces glucosylceramide in the brain. J Lipid Res. 2012;53:282–91. 22058426 10.1194/jlr.M021261 PMC3269155

[10] European Medicines Agency (EMA). European technical data sheet of Cerdelga, INN-eliglustat [Internet]. Available from: https://www.ema.europa.eu/en/documents/product-information/cerdelga-epar-product-information_en.pdf

[11] European Medicines Agency (EMA). European technical data sheet of Yargesa, INN-miglustat [Internet]. Available from: https://www.ema.europa.eu/en/documents/product-information/yargesa-epar-product-information_en.pdf

[12] European Medicines Agency (EMA). European technical data sheet of Miglustat Dipharma 100 mg capsules [Internet]. Available from: https://www.ema.europa.eu/en/documents/product-information/miglustat-dipharma-epar-product-information_en.pdf

[13] European Medicines Agency (EMA). European technical data sheet of Zavesca, INN-miglustat [Internet]. Available from: https://www.ema.europa.eu/en/documents/product-information/zavesca-epar-product-information_en.pdf

[14] European Medicines Agency (EMA). European technical data sheet of Cerezyme, INN-imiglucerase [Internet]. Available from: https://www.ema.europa.eu/en/documents/product-information/cerezyme-epar-product-information_en.pdf

[15] European Medicines Agency (EMA). European technical data sheet of VPRIV^®^, INN-velaglucerase alfa [Internet]. Available from: https://www.ema.europa.eu/en/documents/product-information/vpriv-epar-product-information_en.pdf

[16] Elstein D, Belmatoug N, Bembi B, Deegan P, Fernandez-Sasso D, Giraldo P, et al. Twelve years of the Gaucher outcomes survey (GOS): Insights, Achievements, and lessons learned from a global patient registry. JCM. 2024;13:3588. 38930117 10.3390/jcm13123588 PMC11204885

[17] Bennett LL, Fellner C. Pharmacotherapy of Gaucher disease: current and future options. P T. 2018;43:274–309. 29719368 PMC5912244

[18] Gupta P, Pastores G. Pharmacological treatment of pediatric Gaucher disease. Expert Rev Clin Pharmacol. 2018;11:1183–94. 30444430 10.1080/17512433.2018.1549486

[19] Andrade-Campos MM, De Frutos LL, Cebolla JJ, Serrano-Gonzalo I, Medrano-Engay B, Roca-Espiau M, et al. Identification of risk features for complication in Gaucher’s disease patients: a machine learning analysis of the Spanish registry of Gaucher disease. Orphanet J Rare Dis. 2020;15:256. 32962737 10.1186/s13023-020-01520-7 PMC7507684

[20] Kaplan P, Baris H, De Meirleir L, Di Rocco M, El-Beshlawy A, Huemer M, et al. Revised recommendations for the management of Gaucher disease in children. Eur J Pediatr. 2013;172:447–58. 22772880 10.1007/s00431-012-1771-z

[21] Page MJ, McKenzie JE, Bossuyt PM, Boutron I, Hoffmann TC, Mulrow CD, et al. The PRISMA 2020 statement: an updated guideline for reporting systematic reviews. BMJ. 2021;372:n71. 10.1136/bmj.n71 PMC8005924 33782057

[22] Society for the Study of Inborn Errors of Metabolism (SSIEM) [Internet]. Available from: https://www.ssiem.org/

[23] WORLDSymposium [Internet]. Available from: https://www.worldsymposia.org/

[24] Society for Inherited Metabolic. Disorders (SIMD) [Internet]. Available from: https://www.simd.org/

[25] Basiri M, Ghaffari ME, Ruan J, Murugesan V, Kleytman N, Belinsky G, et al. Osteonecrosis in Gaucher disease in the era of multiple therapies: biomarker set for risk stratification from a tertiary referral center. eLife. 2023;12:e87537. 37249220 10.7554/eLife.87537 PMC10317498

[26] Becker-Cohen M, Revel‐Vilk S, Frydman D, Dinur T, Tiomkin M, Istaiti M, et al. Rapid home therapy infusion of velaglucerase alfa in naïve patients with Gaucher disease. Internal Medicine Journal. 2023;imj.16179. 10.1111/imj.16179 37493453

[27] Stiles AR, Huggins E, Fierro L, Jung S-H, Balwani M, Kishnani PS. The role of glucosylsphingosine as an early indicator of disease progression in early symptomatic type 1 Gaucher disease. Mol Genet Metabolism Rep. 2021;27:100729. 10.1016/j.ymgmr.2021.100729 PMC7876627 33614410

[28] Soudek L, Siddiqui I, Guerin A, Sondheimer N, Inbar-Feigenberg M, Abuquteish D, et al. Liver transplantation for Gaucher disease presenting as neonatal cholestasis: case report and literature review. Pediatr Transplant. 2020;24:e13718. 32324335 10.1111/petr.13718

[29] Schwartz IVD, Göker-Alpan Ö, Kishnani PS, Zimran A, Renault L, Panahloo Z, et al. Characteristics of 26 patients with type 3 Gaucher disease: A descriptive analysis from the Gaucher outcome survey. Mol Genet Metabolism Rep. 2018;14:73–9. 10.1016/j.ymgmr.2017.10.011 PMC5758841 29326879

[30] Tantawy AAG, El-Beshlawy A, Marzouk I, Bavdekar A, Qin Y, Mellgard B, et al. Results from a 12-month open-label phase 1/2 study of velaglucerase alfa in children and adolescents with type 3 Gaucher disease. J Inborn Errors Metabolism Screen. 2018;6:232640981876556.

[31] Zimran A, Elstein D, Gonzalez DE, Lukina EA, Qin Y, Dinh Q, et al. Treatment-naïve Gaucher disease patients achieve therapeutic goals and normalization with velaglucerase Alfa by 4 years in phase 3 trials. Blood Cells Molecules Dis. 2018;68:153–9. 10.1016/j.bcmd.2016.10.007 27839979

[32] Ida H, Tanaka A, Matsubayashi T, Murayama K, Hongo T, Lee H-M, et al. A multicenter, open-label extension study of velaglucerase Alfa in Japanese patients with Gaucher disease: results after a cumulative treatment period of 24 months. Blood Cells Molecules Dis. 2016;59:140–7. 10.1016/j.bcmd.2015.10.002 27241455

[33] Pastores GM, Turkia HB, Gonzalez DE, Ida H, Tantawy AAG, Qin Y, et al. Development of anti-velaglucerase Alfa antibodies in clinical trial-treated patients with Gaucher disease. Blood Cells Molecules Dis. 2016;59:37–43. 10.1016/j.bcmd.2016.03.004 27282565

[34] Smith L, Rhead W, Charrow J, Shankar SP, Bavdekar A, Longo N, et al. Long-term velaglucerase Alfa treatment in children with Gaucher disease type 1 naïve to enzyme replacement therapy or previously treated with imiglucerase. Mol Genet Metab. 2016;117:164–71. 26043810 10.1016/j.ymgme.2015.05.012

[35] Turkia HB, Gonzalez DE, Barton NW, Zimran A, Kabra M, Lukina EA, et al. Velaglucerase Alfa enzyme replacement therapy compared with imiglucerase in patients with Gaucher disease. Am J Hematol. 2013;88:179–84. 23400823 10.1002/ajh.23382

[36] Vairo F, Netto C, Dorneles A, Mittelstadt S, Wilke M, Doneda D, et al. Enzyme replacement therapy in a patient with Gaucher disease type III: a paradigmatic case showing severe adverse reactions started a long time after the beginning of treatment. In: Zschocke J, Gibson KM, Brown G, Morava E, Peters V, editors. JIMD Reports - Volume 11 [Internet]. Berlin, Heidelberg: Springer Berlin Heidelberg; 2013. pp. 1–6. Available from: 10.1007/8904_2013_214. 10.1007/8904_2013_214 PMC3755564 23430813

[37] Zimran A, Pastores GM, Tylki-Szymanska A, Hughes DA, Elstein D, Mardach R, et al. Safety and efficacy of velaglucerase Alfa in Gaucher disease type 1 patients previously treated with imiglucerase. Am J Hematol. 2013;88:172–8. 23339116 10.1002/ajh.23383 PMC3586535

[38] Goker-Alpan O, Khan N, Friedman A, Ivanova M, Pathak R, Wright E. Treatment of infants and very young children with Gaucher disease with velaglucerase alfa: a single-center experience. J Inherit Metab Dis. 2023;46(S1):319.

[39] Soudek L, Siddiqui I, Guerin A, Sondheimer N, Kamath BM, Walia J, et al. Tu1547 – Gaucher disease presenting as neonatal cholestasis with absence of neurological involvement. Case Report & Literature Review. Gastroenterology. 2019. p. 1052.

[40] Elstein D, Zimran A, Gonzalez DE, Lukina EA, Qin Y, Dinh Q, et al. Therapeutic goals and normal clinical values achieved within 4 years of initiating velaglucerase alfa in treatment-naÃ¯ve patients with Gaucher disease in phase 3 studies. Molecular Genetics and Metabolism; 2016. p. 44.

[41] Tantawy AAG, El-Beshlawy A, Marzouk I, Bavdekar A, Qin Y, Mellgard B, et al. Velaglucerase Alfa enzyme replacement therapy in children and adolescents with type 3 Gaucher disease: results of a 12-month multicenter, open-label phase 1/2 study. Molecular Genetics and Metabolism; 2016. p. 111.

[42] Bailey L, Ambruso D, Grabowski G, Burrow TA. Combination therapy (eliglustat + velaglucerase alfa) in a pediatric patient with Gaucher disease type 1 and hereditary spherocytosis. Molecular Genetics and Metabolism; 2015. p. 17.

[43] Pastores G, Turkia H, Gonzalez DE, Ida H, Tantawy A, Lee H, et al. Development of anti-drug antibodies in Gaucher disease patients treated in velaglucerase alfa clinical trials. Blood Cells Mol Dis. 2016:59:37–43. 10.1016/j.bcmd.2016.03.004 27282565

[44] Pastores G, Hendrisksz C, Giugliani R, Braunlin E, Quartel A. Long-term velaglucerase alfa enzyme replacement therapy in children with type 1 Gaucher disease. J Inherit Metab Dis. 2014;37(S1):S153. 10.1007/s10545-014-9693-8 24638276

[45] Giraldo P, Smith L, Harmatz P, Zahrieh D, Crombez E, Cohn G. Exploratory assessment of growth and bone marrow burden in a paediatric subgroup with type 1 Gaucher disease transitioned from imiglucerase to velaglucerase alfa. J Inherit Metab Dis. 2013;36(S2):S302.

[46] Zimran A, Kishnani P, Elstein D, Gonzalez DE, Zahrieh D, Crombez E, et al. Exploratory assessment of growth and bone marrow burden in a pooled subgroup of paediatric patients with type 1 Gaucher disease treated with long-term velaglucerase alfa. J Inherit Metab Dis. 2013;36(S2):S302

[47] Zimran A, Gonzalez DE, Elstein D, Crombez E, Bhirangi K. 1261 enzyme replacement therapy with velaglucerase Alfa improves key clinical parameters in a pediatric subgroup with type 1 Gaucher disease. Pediatric Research. 2010. p. 625.

[48] Leonart LP, Fachi MM, Böger B, da Silva MR, Szpak R, Lombardi NF, et al. A systematic review and meta-analyses of longitudinal studies on drug treatments for Gaucher disease. Ann Pharmacother. 2023;57:267–82. 35815393 10.1177/10600280221108443

[49] Jones SA, Rojas-Caro S, Quinn AG, Friedman M, Marulkar S, Ezgu F, et al. Survival in infants treated with sebelipase alfa for lysosomal acid lipase deficiency: an open-label, multicenter, dose-escalation study. Orphanet J Rare Dis. 2017;12:25. 28179030 10.1186/s13023-017-0587-3 PMC5299659

[50] Kishnani PS, Kronn D, Brassier A, Broomfield A, Davison J, Hahn SH, et al. Safety and efficacy of avalglucosidase alfa in individuals with infantile-onset Pompe disease enrolled in the phase 2, open-label Mini-COMET study: the 6-month primary analysis report. Genet Sci. 2023;25:100328. 10.1016/j.gim.2022.10.010 36542086

[51] Giuffrida G, Markovic U, Condorelli A, Calafiore V, Nicolosi D, Calagna M, et al. Glucosylsphingosine (Lyso-Gb1) as a reliable biomarker in Gaucher disease: a narrative review. Orphanet J Rare Dis. 2023;18:27. 36782327 10.1186/s13023-023-02623-7 PMC9926807

[52] Dinur T, Bauer P, Beetz C, Cozma C, Becker-Cohen M, Istaiti M, et al. Contribution of glucosylsphingosine (Lyso-Gb1) to treatment decisions in patients with Gaucher disease. IJMS. 2023;24:3945. 36835356 10.3390/ijms24043945 PMC9966520

[53] Tatiana Raskovalova PB, Deegan PK, Mistry E, Pavlova R, Yang A, Zimran, et al. Accuracy of Chitotriosidase activity and CCL18 concentration in assessing type I Gaucher disease severity. A systematic review with meta-analysis of individual participant data. Haematol. 2020;106:437–45. 10.3324/haematol.2019.236083 PMC7849573 32001533

[54] Slade A, Isa F, Kyte D, Pankhurst T, Kerecuk L, Ferguson J, et al. Patient reported outcome measures in rare diseases: a narrative review. Orphanet J Rare Dis. 2018;13:61. 29688860 10.1186/s13023-018-0810-x PMC5914068

[55] Cerón-Rodríguez M, Barajas‐Colón E, Ramírez‐Devars L, Gutiérrez‐Camacho C, Salgado‐Loza JL. Improvement of life quality measured by Lansky score after enzymatic replacement therapy in children with Gaucher disease type 1. Molec Gen Gen Med. 2018;6:27–34. 10.1002/mgg3.339 PMC5823673 29471591

[56] Feng J, Gao Z, Shi Z, Wang Y, Li S. Patient-reported outcomes in Gaucher’s disease: a systematic review. Orphanet J Rare Dis. 2023;18:244. 37626429 10.1186/s13023-023-02844-w PMC10463869

[57] McDool E, Powell P, Carlton J. Measuring health related quality of life (HRQoL) in lysosomal storage disorders (LSDs): a rapid scoping review of available tools and domains. Orphanet J Rare Dis. 2024;19:252. 38965628 10.1186/s13023-024-03256-0 PMC11225496

[58] Elstein D, Belmatoug N, Deegan P, Göker-Alpan Ö, Hughes DA, Schwartz IVD, et al. Development and validation of Gaucher disease type 1 (GD1)-specific patient-reported outcome measures (PROMs) for clinical monitoring and for clinical trials. Orphanet J Rare Dis. 2022;17:9. 34991656 10.1186/s13023-021-02163-y PMC8734239

[59] Germain N, Aballéa S, Toumi M. Measuring health-related quality of life in young children: how Far have we come? J Market Access Health Policy. 2019;7:1618661. 10.1080/20016689.2019.1618661 PMC6534256 31156762

[60] Revel-Vilk S, Mansfield R, Feder-Krengel N, Machtiger-Azoulay N, Kuter D, Szer J, et al. Real-World experiences with taliglucerase Alfa home infusions for patients with Gaucher disease: A global cohort study. JCM. 2023;12:5913. 37762854 10.3390/jcm12185913 PMC10531841

[61] Brunelli MV, Rabhansl MM, Delacre C, Dankert MM, Cuevillas MV, Frias CT. Home-Based care for patients with lysosomal storage disease: experiences in Argentina. J Inborn Errors Metab Screen. 2019;7:e20180002.

[62] Polinski JM, Kowal MK, Gagnon M, Brennan TA, Shrank WH. Home infusion: Safe, clinically effective, patient preferred, and cost saving. Healthcare. 2017;5:68–80. 28668202 10.1016/j.hjdsi.2016.04.004

[63] Elstein D, Abrahamov A, Oz A, Arbel N, Baris H, Zimran A. 13,845 home therapy infusions with velaglucerase Alfa exemplify safety of velaglucerase Alfa and increased compliance to every-other-week intravenous enzyme replacement therapy for Gaucher disease. Blood Cells Molecules Dis. 2015;55:415–8. 10.1016/j.bcmd.2015.09.002 26460268

[64] Elstein D, Burrow TA, Charrow J, Giraldo P, Mehta A, Pastores GM, et al. Home infusion of intravenous velaglucerase alfa: experience from pooled clinical studies in 104 patients with type 1 Gaucher disease. Mol Genet Metab. 2017;120:111–5. 27614581 10.1016/j.ymgme.2016.08.005

[65] Zimran A, Wang N, Ogg C, Crombez E, Cohn GM, Elstein D. Seven-year safety and efficacy with velaglucerase Alfa for treatment‐naïve adult patients with type 1 G Aucher disease. Am J Hematol. 2015;90:577–83. 25903392 10.1002/ajh.24040 PMC5033020

[66] Heinrich R, Claus F, Schoenfelder T. The patients` perspective on home-based infusion: a longitudinal observational study in the German healthcare setting for patients with lysosomal storage disorders treated with enzyme replacement therapy. Mol Genet Metabolism Rep. 2023;35:100971. 10.1016/j.ymgmr.2023.100971 PMC10090431 37065272

[67] Andrade-Campos M, Escuder-Azuara B, De Frutos LL, Serrano-Gonzalo I, Giraldo P. Direct and indirect effects of the SARS-CoV-2 pandemic on Gaucher disease patients in spain: time to reconsider home-based therapies? Blood Cells Molecules Dis. 2020;85:102478. 10.1016/j.bcmd.2020.102478 PMC7358160 32688219

[68] Vellodi A, Tylki-Szymanska A, Davies EH, Kolodny E, Bembi B, Collin‐Histed T, et al. Management of neuronopathic Gaucher disease: revised recommendations. J Inher Metab Disea. 2009;32:660–4. 10.1007/s10545-009-1164-2 19655269

[69] Deegan P, Lau H, Elstein D, Fernandez-Sasso D, Giraldo P, Hughes D, et al. Long-Term treatment of Gaucher disease with velaglucerase Alfa in ERT-Naïve patients from the Gaucher outcome survey (GOS) registry. JCM. 2024;13:2782. 38792324 10.3390/jcm13102782 PMC11122485

[70] El-Beshlawy A, Tylki-Szymanska A, Vellodi A, Belmatoug N, Grabowski GA, Kolodny EH, et al. Long-term hematological, visceral, and growth outcomes in children with Gaucher disease type 3 treated with imiglucerase in the international collaborative Gaucher group Gaucher registry. Mol Genet Metab. 2017;120:47–56. 28040394 10.1016/j.ymgme.2016.12.001

[71] Altarescu G, Hill S, Wiggs E, Jeffries N, Kreps C, Parker CC, et al. The efficacy of enzyme replacement therapy in patients with chronic neuronopathic Gaucher’s disease. J Pediatr. 2001;138:539–47. 11295718 10.1067/mpd.2001.112171

[72] Grabowski GA, Antommaria AHM, Kolodny EH, Mistry PK. Gaucher disease: basic and translational science needs for more complete therapy and management. Mol Genet Metab. 2021;132:59–75. 33419694 10.1016/j.ymgme.2020.12.291 PMC8809485

[73] Irún P, Alfonso P, Aznarez S, Giraldo P, Pocovi M. Chitotriosidase variants in patients with Gaucher disease. Implications for diagnosis and therapeutic monitoring. Clin Biochem. 2013;46:1804–7. 24060732 10.1016/j.clinbiochem.2013.09.006

