# Supplementary material for: Investigating the therapeutic profile of velaglucerase alfa in paediatric patients with Gaucher disease: a systematic review across all paediatric age groups
Source: Orphanet J Rare Dis. 2026 Feb 5;21:91. doi: 10.1186/s13023-026-04221-9 (PMC12973762; doi:10.1186/s13023-026-04221-9)
Supplement: Supplementary file 1 — Supplementary Material 1 [file 13023_2026_4221_MOESM1_ESM.docx]

**SUPPLEMENTARY MATERIAL**

**Unknown GD type**

In total, 4 (17.39%, n=16) studies were identified that investigated the effectiveness of velaglucerase alfa in treating paediatric patients with GD of unknown or unspecified type (Table S1) [25,28,38,39]. Haematological parameters were described in 3 (13.04%, n=15) publications, and they either increased or remained stable in those patients [25,28,38]. Additionally, liver and spleen volumes, which were reported in 3 (13.04%, n=15) studies, also remained stable or increased in all patients except one, in which massive hepatosplenomegaly did not improve [25,38,39]. Information related to bone parameters was reported in 2 (8.69%, n=14) articles. Bone pain was reported by 2 patients [25], and height percentiles improved or remained stable [38]. Plasma biomarkers were included in 3 (13.04%, n=15) studies. The ChT activity of GD paediatric patients remained stable in one of the studies [25] and increased in another study, although this increase was followed by a partial reduction [28]. Additionally, a reduction in Lyso-Gb1 concentration after velaglucerase alfa treatment was observed [25,38].

Only one (4.35%, n=12) study included information about the safety of velaglucerase alfa treatment. Non-TEAEs were reported in this study [38].

**Table S1. Patient efficacy observations. Unspecified GD type**

| **Author, year**  **[reference]** | **Patients’ description** | **Haematological parameters** | **Visceral parameters** | **Bone parameters** | **Plasma biomarkers** | **Quality of life** |
| --- | --- | --- | --- | --- | --- | --- |
| **Basiri, 2023 [25]** | 2 naïve patients with GD | The Hb concentration was 11.5–13.4 g/dL, and the platelet count was 124–212 × 1000 u/L. | The liver and spleen volumes were 1.12–1.38 MN and 3.1–7.7 MN, respectively. | Both patients reported bone pain. | The ChT activity was 731.4–4134.6 nmol/h/mL, and the Lyso-Gb1 concentration was 143.5–318.6 ng/mL. | ND |
| **Goker-Alpan, 2023 [38]** | 12 patients with GD1 or GD3 | The Hb concentration (baseline: 10.6–12.4 g/dL) and platelet count (baseline: 113–371 × 10^9^/L) increased or remained stable in all patients. | The liver and spleen volumes increased or remained stable in all patients. | Growth parameters and percentiles increased or remained stable in most patients. | Within the first 6 months of treatment, the Lyso-Gb1 concentration decreased from 90–874 to 3.8–26 ng/mL. | ND |
| **Soudek, 2020 [28]** | 1 naïve patient with GD***** | The Hb concentration increased from 94 to 100 g/L, and the platelet count increased from 33 × 10^9^/L to 88 × 10^9^/L, not reaching normal values. | ND | ND | The ChT activity initially increased but partially decreased afterwards. | ND |
| **Soudek, 2019 [39]** | 1 naïve patient with GD | ND | After 2 months, massive hepatosplenomegaly was still observed. | ND | ND | ND |

ChT: Chitotriosidase; GD: Gaucher disease; Hb: Haemoglobin; Lyso-Gb1: Glucosylsphingosine; MN: Multiples of normal

* Patient died at 8 months of age.
